# Positive impact of preventative chemotherapy during a national helminth control program: Perception and KAP

**DOI:** 10.1371/journal.pntd.0008494

**Published:** 2020-08-24

**Authors:** Francisca Mutapi, Paradzayi Tagwireyi, Rivka Lim, Blessing Mangwanda, Charmaine Fourier, Takafira Mduluza

**Affiliations:** 1 Institute of Immunology & Infection Research, University of Edinburgh, Ashworth Laboratories, Edinburgh, United Kingdom; 2 NIHR Global Health Research Unit Tackling Infections to Benefit Africa (TIBA) at the University of Edinburgh, Ashworth Laboratories, Edinburgh, United Kingdom; 3 Department of Geography and Environmental Science, Geo-information and Earth Observation Centre, University of Zimbabwe, Mount Pleasant, Harare, Zimbabwe; 4 Department of Biochemistry, University of Zimbabwe, Mount Pleasant, Harare, Zimbabwe; Federal University of Ceará, Fortaleza, Brazil, BRAZIL

## Abstract

Helminth control at the national level is currently based on mass drug administration (MDA) programs. Perception of the MDA programs for helminth control by the affected populations influences compliance and future designs of the programs. We determined the perception of Zimbabwe’s National Helminth Control Program (2012–2017) with a specific focus on schistosomiasis in the school children treated with praziquantel, schoolteachers and village health workers (VHW). The study enrolled 409 children from Grades 6 and 7 who had the full benefit of the 6 years of MDA from 2012 to 2017. Thirty-six schoolteachers and 22 VHW serving the schools were also recruited. A structured questionnaire developed in English, translated into the local language Shona, and validated prior to the study was administered to the children and the adults. The questions focused on the perceived impact on health, school attendance and performance and Knowledge Attitudes and Practice (KAP) among the school children. Data were captured electronically on android platforms using the Open Data Kit. Overall, 84% of the children responded that their awareness of schistosomiasis (transmission, disease, treatment and infection avoidance) had improved because of participating in the MDAs. Of the 151 children self-diagnosed with schistosomiasis, 74% reported that their health had improved following treatment with praziquantel. This included resolution of haematuria, painful urination, sore stomach, tiredness and falling asleep during class lessons. The children and teachers reported improvements in both pupil school attendance and performance at school while the VHW and teachers reported an increase in health-seeking behaviour amongst the school children for schistosomiasis treatment in-between MDAs. The majority of VHW (96%) reported improvement in handwashing behaviour, schistosomiasis awareness (96%) and treatment uptake (91%) within the communities where the school children belonged. However, only 59% of the VHW reported improvement in toilet use while only 50% of the VHW reported improvement in clean water use within their communities. This study indicated that the surveyed children perceived the MDA program had improved their health, school attendance, school performance and awareness of schistosomiasis. The VHW also perceived that the MDA program had improved the community KAP.

## Author summary

Perception of mass drug administration (MDA) programs for helminth control by the affected populations influences compliance and therefore affects the success of the program. We determined the perception of the impact of Zimbabwe’s National Helminth Control Program with a specific focus on schistosomiasis in the school children treated with praziquantel and the opinion of schoolteachers and village health workers (VHW). Overall, 84% of the children responded that their awareness of schistosomiasis had improved because of participating in the MDAs. Of the children self-diagnosed with schistosomiasis, over 90% reported that their health had improved following treatment with praziquantel. This included resolution of self identified health symptoms including haematuria, painful urination, sore stomach, tiredness and falling asleep during class lessons, although the latter two were less frequent before treatment. The children and teachers reported improvements in both pupil school attendance and performance at school as well as increased health-seeking behaviour for schistosomiasis treatment in-between MDAs. Finally, > 90% of the VHW reported improvement in handwashing behaviour, schistosomiasis awareness and treatment uptake within the community. However, only 59% of the VHW reported improvement in toilet use while only 50% of the VHW reported improvement in clean water use, within their communities, highlighting the need for improved safe water and sanitation provision.

## Introduction

Public health interventions rely on the compliance of the target population for efficacy and success. In the case of helminth control programs which rely on mass drug administration (MDA) to treat exposed people, drug treatment in areas with high infection prevalence can occur annually for several years [1], requiring community compliance to repeated treatment. Understanding the community perception of schistosomiasis interventions is particularly important to policy makers and program implementers [2]. Experience from schistosomiasis control in Brazil and Uganda highlights the importance of feedback and continued evaluation of the acceptance and response to introduced control programs [3, 4].

This study was conducted in Zimbabwe, a country endemic for several of the neglected tropical diseases (NTDs) as listed by the World Health Organization (WHO) [5]. Of these NTDs, schistosomiasis (both urogenital and intestinal) is the most prevalent in the country [6]. In 2012, the WHO set out a roadmap for NTDs, including controlling schistosomiasis morbidity by 2020, elimination of schistosomiasis as a public health problem and interrupting transmission in various African countries by 2025 [7]. As part of implementing this roadmap, the ministry of health in Zimbabwe implemented a national helminth control program from 2012 to 2017, which targeted all primary school children [8]. In this control program, children were treated for soil-transmitted helminths and schistosomes through annual mass drug administration of albendazole and praziquantel respectively. We have investigated the impact of the national control program on schistosome infection and morbidity levels in a cohort of children followed for 6 years [9] and a national program impact assessment was recently performed. The cohort study has indicated that Zimbabwe’s MDA has significantly reduced schistosome infection intensity and prevalence and urogenital schistosomiasis morbidity prevalence in the school-aged children, moving the schistosome prevalence in the children from moderate to low by WHO classification. However to date, there had been no assessment of the patient experience, the perceptions of children and population who received MDAs or adults involved in the operational aspects of the MDA.

Therefore, in this study, we aimed to interview treated children, their Village Health Workers (VHW) and their teachers on their experience of mass drug treatment of schistosomiasis. We focused on their perception of the impact of treatment on their overall health, the side effects of treatment and knowledge attitudes and practices (KAP). While informing Zimbabwe’s next strategy for schistosome control, findings from this study will also be valuable for feeding into a systems epidemiology approach [10] for tackling schistosomiasis in the drive for eliminating it as a public health problem.

## Materials and methods

This study was part of a larger study investigating the impact of Zimbabwe’s national helminth control program using mass drug administration on schistosome infection and morbidity as well as the overall health of treated children.

### Ethics statement

The study received institutional approval from the University of Zimbabwe and Ethical approval from the Medical Research Council of Zimbabwe MRCZ/A/1710. Permission to conduct the study in the province was obtained from the Ministry of Health & Child Care. Before visiting, the school children were given letters inviting one of their parents or guardian to report to the school for the survey at a given date. Before recruitment, the survey was explained to parents/guardians who then gave written consent for the child to participate in the study. On the day of the study, the student had the option to participate or not. The parents/guardians were free to withdraw the participants at any time with no further obligation

### Study area

**Fig 1:**
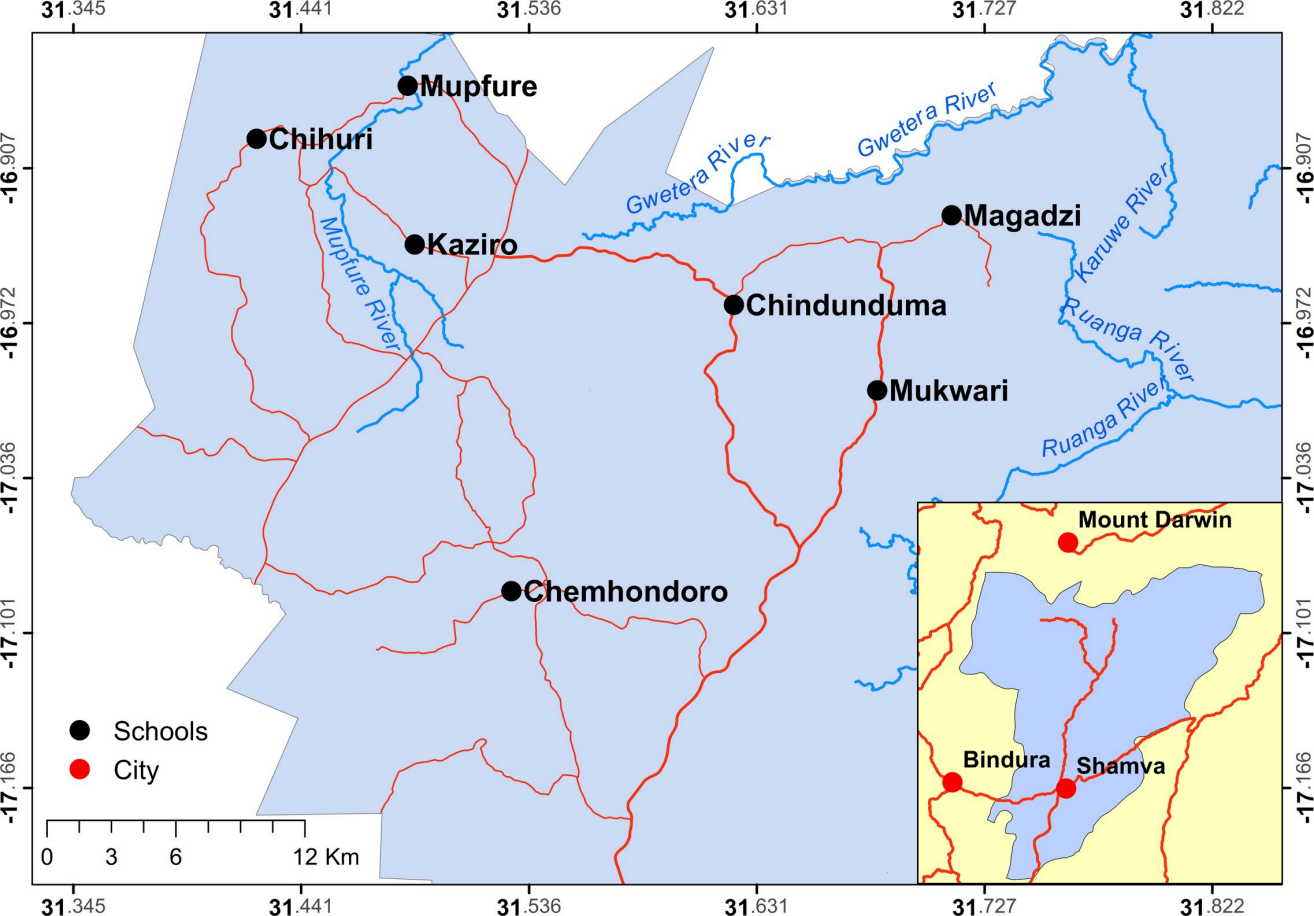
Map of the study area (Madziwa) showing locations of the seven schools enrolled in the study. Map produced using the software package ArcMap 10.1.

The study was conducted in the Madziwa area in Zimbabwe. The study area, Madziwa (17°04′S 31°40′E), was chosen as a previous national survey showed that the area has a high prevalence of schistosomiasis with *S*. *haematobium* being >50% [6]. Prevalences of soil-transmitted helminths and *S*. *mansoni* is low in the area [6]. Seven schools were enrolled in the study and their locations are shown in Fig 1.

### Study schools and participants

The seven primary schools enrolled in this study had received mass drug treatments for schistosomiasis and soil-transmitted helminths during the country’s national helminth control program. Zimbabwe’s MDA was school-based, targeting primary and secondary school children. To capture impact at individual levels, the study focused on primary school children. Primary school children are in grades 1 to 7, and these children have received MDA for 6 years (2012–2017). Therefore, the children in grades 6 and 7 have had the full benefit of the 6 years of MDA from when they were in grades 1 and 2. Thus this survey focused on children in grades 6 and 7 in each school. These were the focus group for assessing the perceived health benefits and KAP following treatment for the helminth infection.

To capture the overall impact in the school and community the questionnaire was also administered to the adults in the schools involved in MDA. When MDA is administered at the school, school teachers and VHW oversee the distribution of treatment and record the MDA in the school MDA register. Therefore, the questionnaire had a section for the school headmaster/mistress and their deputy, the health teacher (every school has one of these) and the VHW (usually 5–10 per school community).

### Inclusion criteria

All children enrolled in this study had to have been involved in the annual MDA as part of the national helminth control program, this was confirmed by the national MDA registers kept by each school. The adults involved had to have been the school headteacher and health teacher(s) or their appointed representative at the time of the MDAs, as well as the VHW serving the communities within the catchment area for the school.

### Sample sizes

For quantifying helminth infection levels in Zimbabwe to inform the national helminth control program, we conducted a national survey where we sampled 50 children from each school based on the sample size calculation previously described [6]. To be consistent, we sampled at least 50 children in each school (25 in grade 6 and 25 in grade 7). In schools where the grade 6 or grade 7 enrolment was low, we sampled all children in those classes giving us a sample size lower than 50. Similarly, in schools where the collective sample size of grade 6 and 7 was between 50 and 70, we sampled all willing children to avoid stigmatizing children not selected.

From each school at least 3 adults (teaching staff and village health worker) were also included in the study. In total, 467 individuals, of which 409 were school pupils, 36 were school authorities and 22 were VHW were included in the study. The number of school authorities and VHW varied depending on the school enrolment and catchment area size. The sampling of the children and adults followed convenience sampling.

### Data collection tools

A structured questionnaire was developed in English and translated into the local language Shona. The questionnaire contained both open and closed-ended questions. All the questions were designated as single or multiple-response and all the data were captured electronically on android platforms, using the freely available open data collection tool Open Data Kit (https://getodk.org/). A proportion of the questions especially those on schistosomiasis symptoms and treatment side effects had been validated in previous studies. Other questions were piloted among some representative participants before administration of the questionnaire for the study. Data quality and consistency was checked by the field team leaders daily.

The study asked school pupils to self-report schistosome infection and symptoms as well as self-declare their perception of the impact of treatment on these symptoms as well as on their school performance and attendance. In addition, the study asked the teachers to give their opinion on the impact of the MDA on performance and school attendance of the children, and the VHW their opinion on health-seeking behaviour. Finally, the study assessed the perceived impact of the MDA on KAP in the pupils, as well as the perceived impact of the KAP, which was learnt by the children through participating in the MDAs, on Water, Sanitation and Hygiene (WASH) practices in the communities the children belonged to.

## Results

The survey interviewed 467 individuals, of which 409 were school pupils, 36 were school authorities and 22 were VHW (Table 1). Disaggregation of the data by gender showed that ~57% of the pupils were females, 44% of the school authorities were female and ~86% of the VHW were female (Table 1).

**Table 1:** Number of participants interviewed per school.

	Pupil	School Authority	Village Health Worker	All
	Total *n* (female/male)	Total *n* (female/male)	Total *n* (female/male)	Total *n* (female/male)
Chemhondoro	56 (26/30)	5 (4/1)	-	**61 (30/31)**
Chihuri	54 (31/23)	5 (-/5)	7 (7/-)	**66 (38/28)**
Chindunduma	68 (39/29)	7 (6/1)	-	**75 (45/30)**
Kaziro	66 (31/35)	5 (1/4)	5 (5/-)	**76 (37/39)**
Magadzi	35 (25/10)	4 (2/2)	1 (1/-)	**40 (28/12)**
Mukwari	61 (44/17)	4 (1/3)	2 (2/-)	**67 (47/20)**
Mupfure	69 (36/33)	6 (2/4)	7 (4/3)	**82 (42/40)**
**Total**	**409 (232/177)**	**36 (16/20)**	**22 (19/3)**	**467 (267/200)**

### Children receiving praziquantel treatment

The questionnaire asked the children how many had received at least one treatment for bilharzia (schistosomiasis) in the past 7 years. This was to confirm the MDA register records. We then determined how many children had self-diagnosed with schistosomiasis since before the last MDA in 2017. Of the 409 children, 151 responded that they had self-diagnosed with schistosomiasis (Table 2). We asked what symptoms they had experienced and they indicated passing blood when urinating, pain during urination, sore stomach, tiredness and falling asleep during class lessons as their schistosomiasis symptoms.

**Table 2:** Pupils who self-diagnosed with schistosomiasis.

School	Infected	Uninfected
Chemhondoro	25	31
Chihuri	19	35
Chindunduma	16	52
Kaziro	22	44
Magadzi	15	20
Mukwari	9	52
Mupfure	45	24
**Total**	**151**	**258**

**Fig 2:**
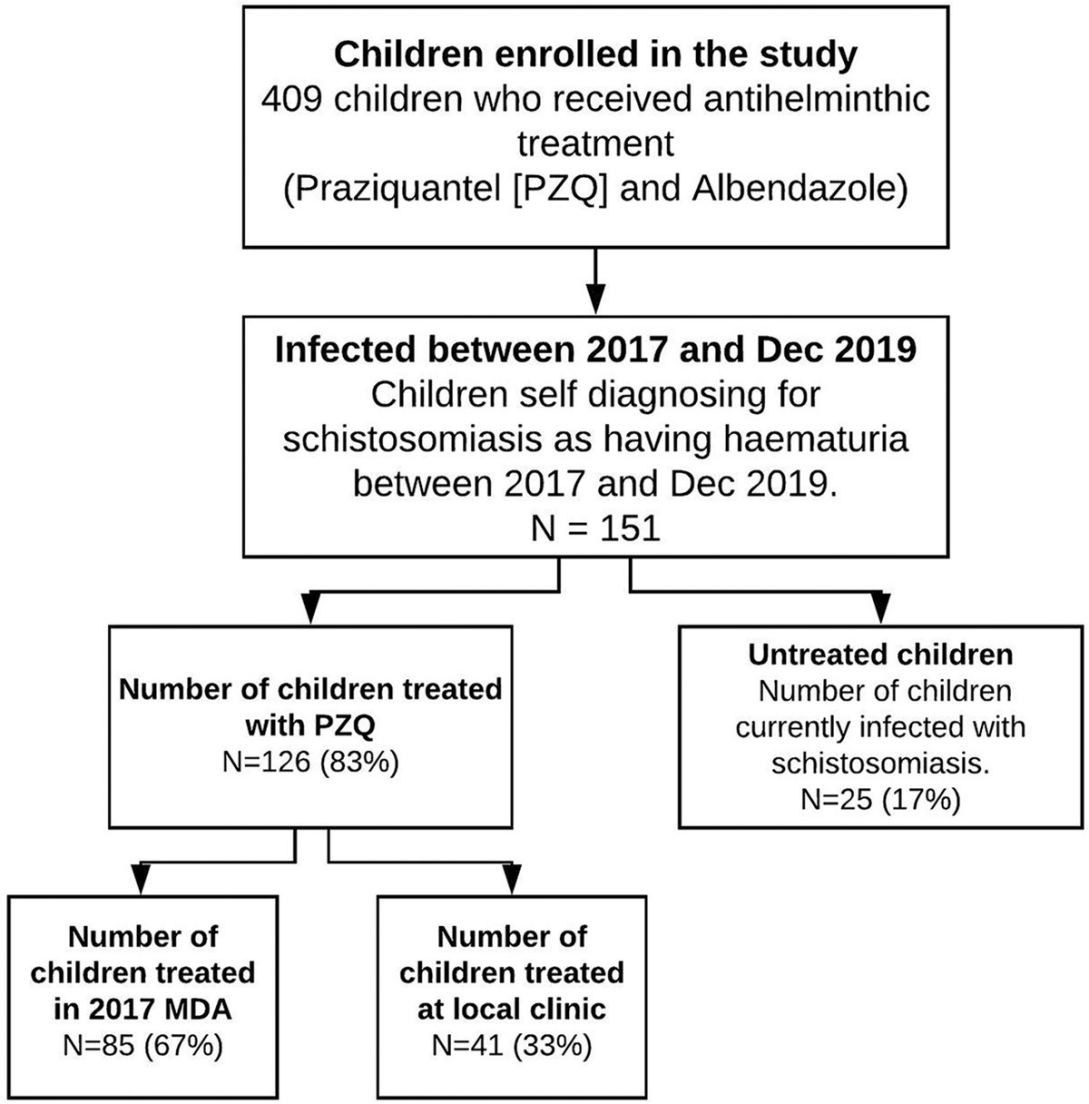
Flow chart of the treatment pathway for the 151 children self-diagnosing for schistosomiasis.

Even though all 151 children had received treatment during the MDAs, some children had subsequently become infected post the last MDA in 2017 and had not been treated at the time of the interview, as the local health centres had reportedly run out of PZQ. The survey showed that 83% of the children (126/151) had been treated for schistosomiasis at the time of the interview. Of the 126 children, 67% (85) of the children were treated during the 2017 MDA while 33% (41) children who had self-diagnosed with schistosomiasis after the 2017 MDA were treated at the local clinic between 2017 (after the 2017 MDA) and 2019 after self-referral for treatment. Twenty-five children (17%) who also self-diagnosed with schistosomiasis during the period between the last MDA and this survey, had not been treated and were still passing blood in their urine. The breakdown of the 151 children by school and treatment status is shown in Table 3. These children have been summarized in Fig 2 below.

**Table 3:** Treatment status of the 151 children self-diagnosing with schistosomiasis at the time of the study.

School	Treated	Untreated
Chemhondoro	18	7
Chihuri	14	5
Chindunduma	14	2
Kaziro	21	1
Magadzi	13	2
Mukwari	8	1
Mupfure	38	7
**Total**	**126**	**25**

**Fig 3:**
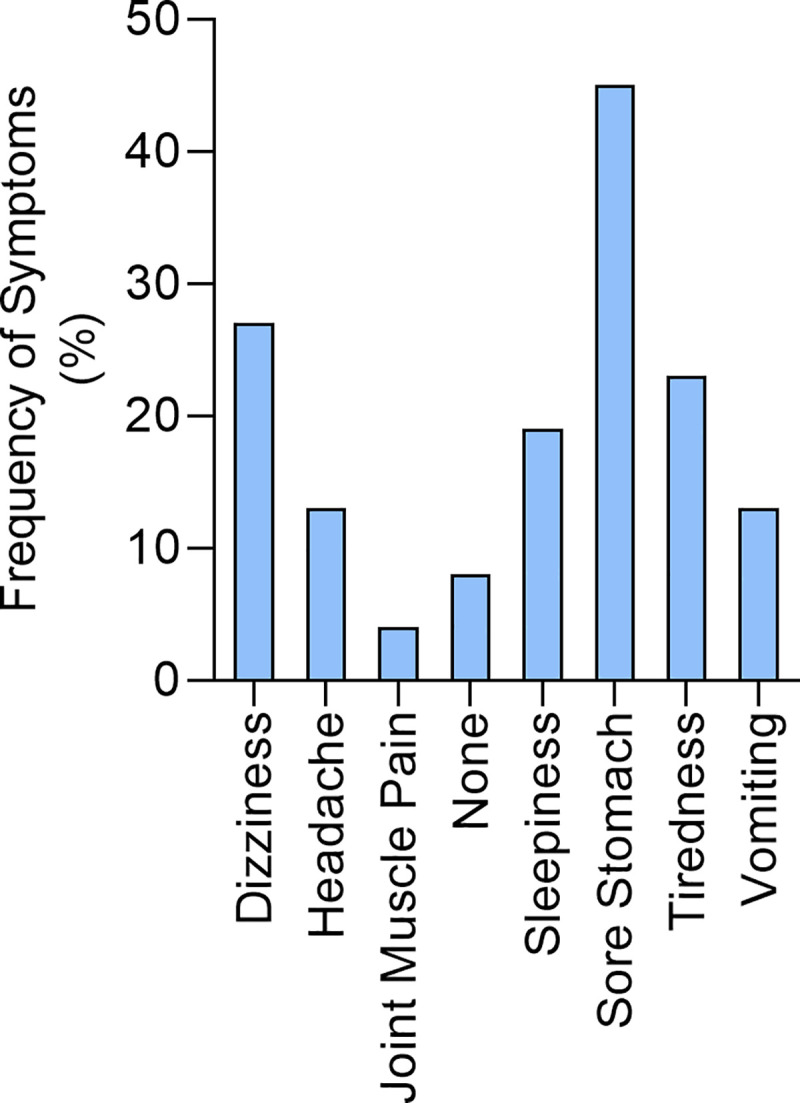
Frequency of side effects following PZQ treatment in children self-diagnosing for schistosomiasis.

Since all 151 children who self-diagnosed with schistosomiasis had received PZQ treatment during the last MDA in 2017, we asked them all to indicate from a list of side effects, which ones they had experienced following treatment for schistosomiasis in 2017. The children identified abdominal pain (sore stomach) as the most frequent side effect (45%) with a few children (8%) experiencing no side effects at all (Fig 3).

**Fig 4:**
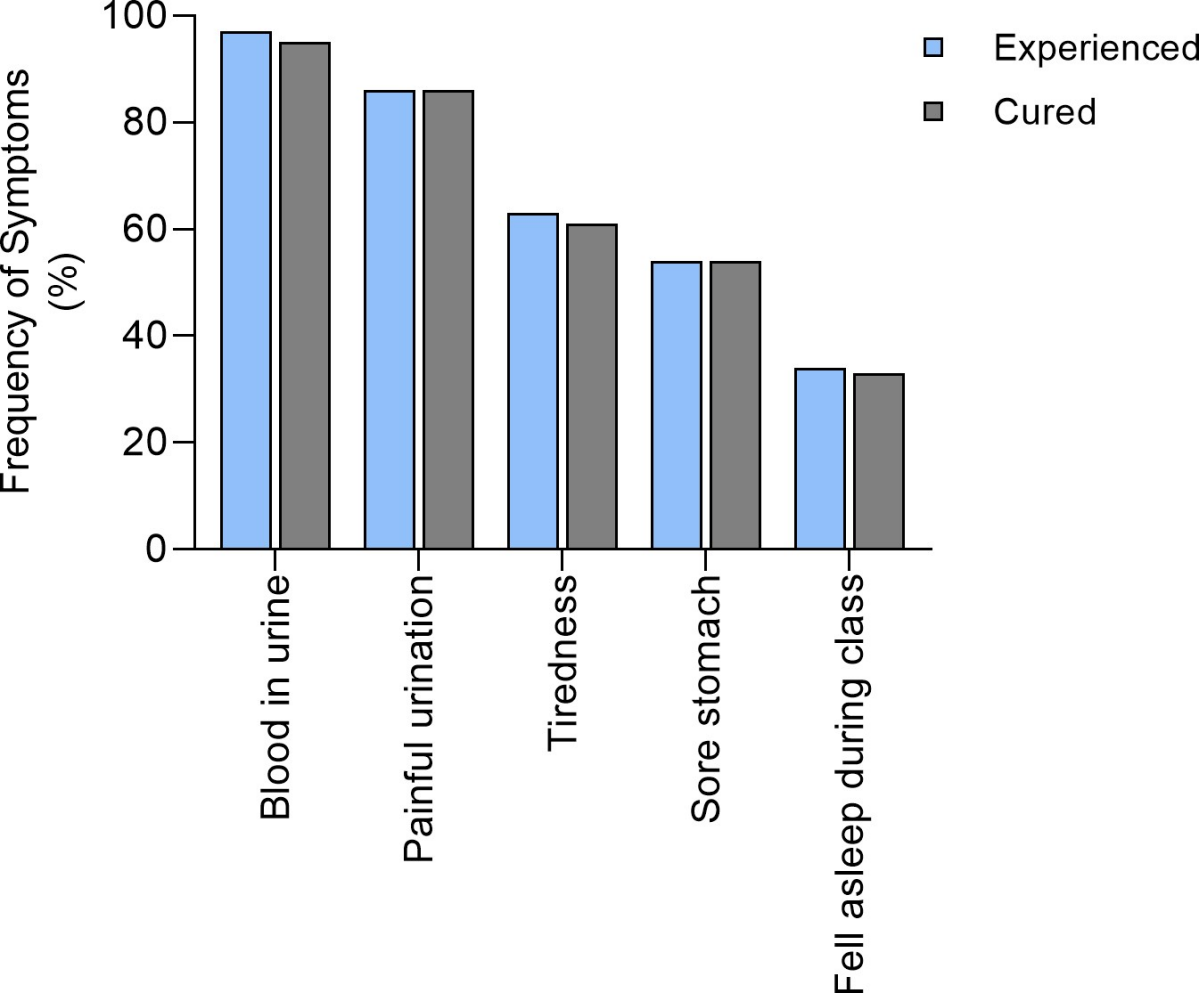
Frequency of symptoms experienced before the treatment and symptoms cured by the treatment amongst the pupils.

We finally asked the 151 children who had self-diagnosed with schistosomiasis if the symptoms they associated with schistosomiasis had resolved/improved after receiving treatment for schistosomiasis during the last MDA in 2017. The children indicated from a list of symptoms, the ones they experienced before schistosomiasis treatment and which resolved/improved following treatment. Haematuria and painful urination were the symptoms most frequently reported by the children. Of the children who reported haematuria and painful urination, 98% and 100% respectively indicated that these symptoms had resolved following treatment (see Fig 4).

### Impact of antihelminthic treatment on school attendance

All the school authorities and all the 151 children who had self-diagnosed with schistosomiasis were asked if the anthelmintic treatment had impacted the number of days they were absent from school, the three possible answers on the questionnaire were that treatment had decreased the number of days children were absent due to illness, had no impact or increased the days they were absent.

**Fig 5:**
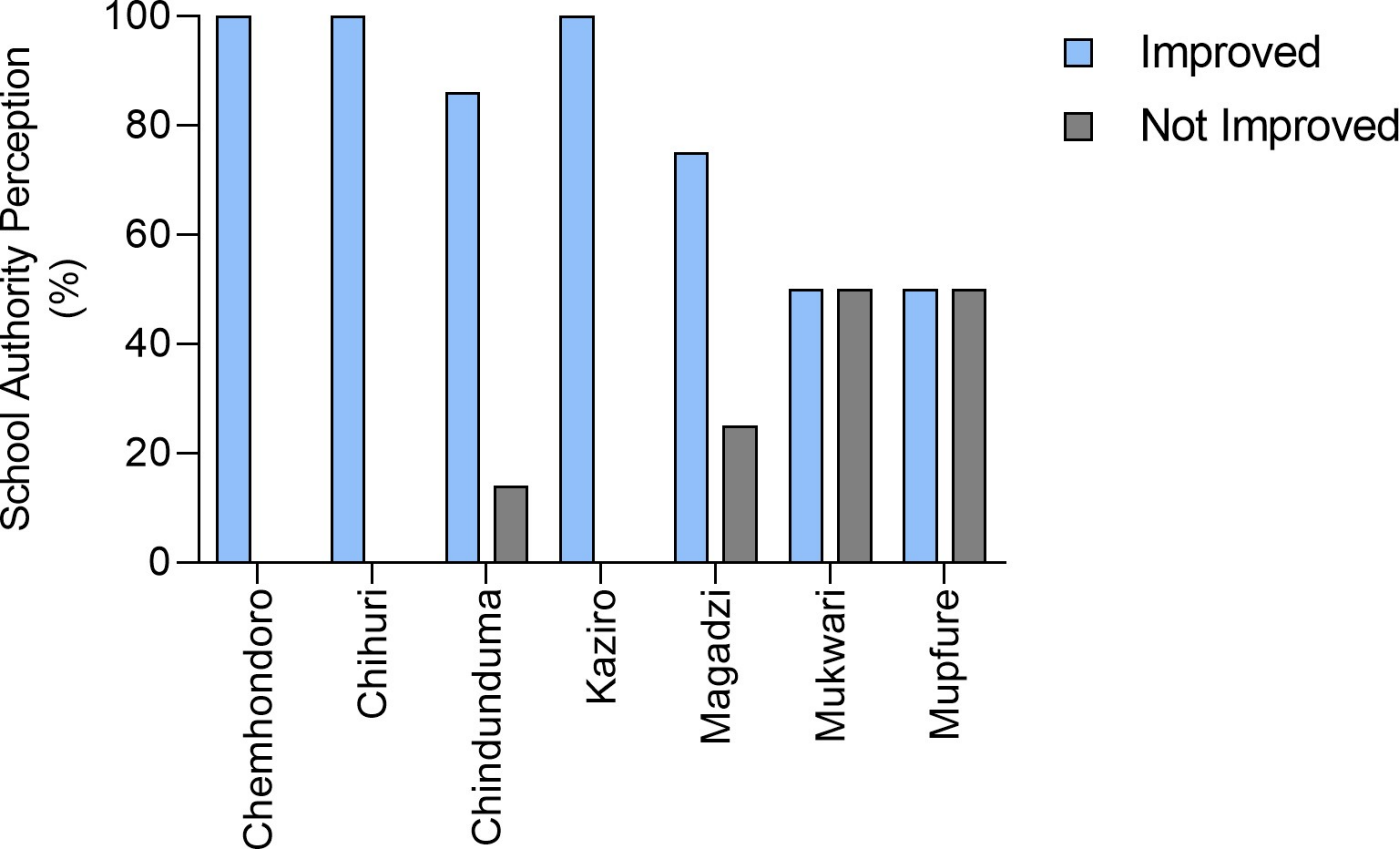
Perception of impact on pupils school attendance by school authorities after treatment.

The pupils self-declared assertions and the adults gave their opinions in response. Looking at the responses from the school teachers, the survey showed that 75% (27) of the school authorities perceived that the national helminth control program intervention had improved school attendance, (Fig 5). In three of the seven schools, Chemhondoro, Chihuri and Kaziro, all teachers responded that school attendance increased after antihelminthic treatment. Noteworthy is that 50% of school authorities at two schools, Mukwari and Mupfure Primary School perceived the intervention as having no effect on school attendance of pupils (Fig 5).

We went on to ask the 151 children who had self-diagnosed for schistosomiasis before antihelminthic treatment, if receiving antihelminthic treatment affected their school performance. Again, the pupils self-declared assertions and the teachers gave their opinions in response. The study showed that 74% (112) of the pupils perceived the effect of their schistosomiasis treatment on their school performance as positive. However, 25% (38) thought that the treatment had no effect on their school performance, whereas one pupil reported that the treatment had a negative effect on their school performance (Table 4).

**Table 4:** Pupils perception of the impact of antihelminthic treatment in their school performance.

School	Improved	No effect	Worsened
Chemhondoro	18	6	1
Chihuri	13	6	-
Chindunduma	15	1	-
Kaziro	8	14	-
Magadzi	15	-	-
Mukwari	5	4	-
Mupfure	38	7	-
**Total**	**112**	**38**	**1**

This finding was consistent with the data from the school authorities of whom 81% (29) perceived the national helminth control program as having a positive impact on the school performance of pupils who were treated for bilharzia (Table 5). All the school authorities from Chemhondoro, Chihuri and Kaziro perceived that the national helminth control program had had a positive effect on pupils’ school performance.

**Table 5:** School authorities’ perception of the impact of antihelminthic treatment on pupil school performance.

School	Improved	No effect
Chemhondoro	5	-
Chihuri	5	-
Chindunduma	6	1
Kaziro	5	-
Magadzi	3	1
Mukwari	2	2
Mupfure	3	3
**Total**	**29**	**7**

### Knowledge attitudes and practices

*Impact of National Helminth Control program on schistosomiasis awareness and reinfection*.

Although the national control program did not have an integrated awareness program, there was a national media awareness campaign before each MDA and also, information about the MDA was given out to pupils and VHW before the MDAs. We determined if the national control program had improved the pupils’ awareness of schistosomiasis. The survey shows that 84% (342) of the pupils responded that their awareness of schistosomiasis had improved as a result of participating in the MDAs. However, noteworthy is the 16% (67) of children who indicated that their awareness of schistosomiasis had not improved over the years of MDA.

**Fig 6:**
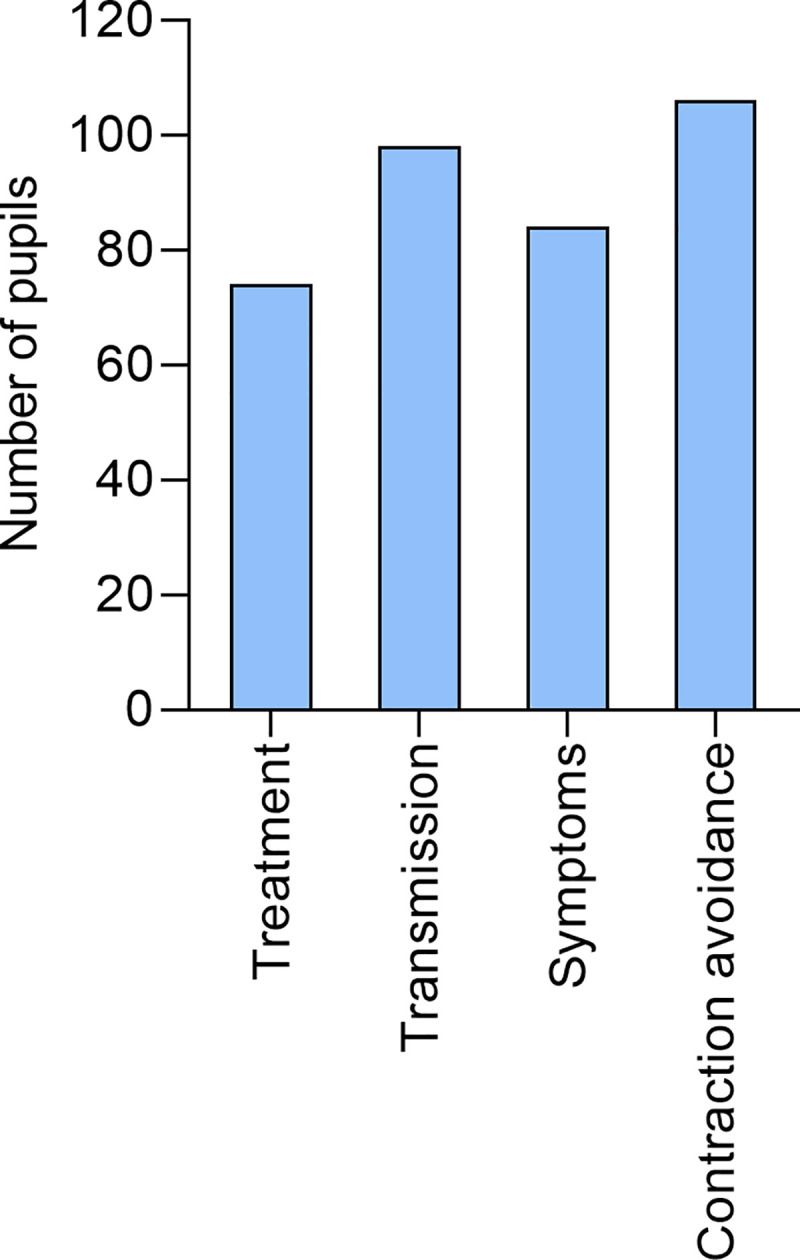
Figure showing the proportion of students who learnt about different aspects of schistosomiasis.

We followed up the question in the 342 children who had responded that their schistosomiasis awareness had improved to determine which specific areas of schistosomiasis awareness, from a list, students’ perception had improved the most over the years of MDA. This included schistosomiasis transmission, disease symptoms, treatment and how to avoid contracting the disease (Fig 6). The largest proportion of children indicated that their understanding of how to avoid contracting schistosomiasis had improved. The least number of children reported that their knowledge of the treatment for the disease had improved.

**Fig 7:**
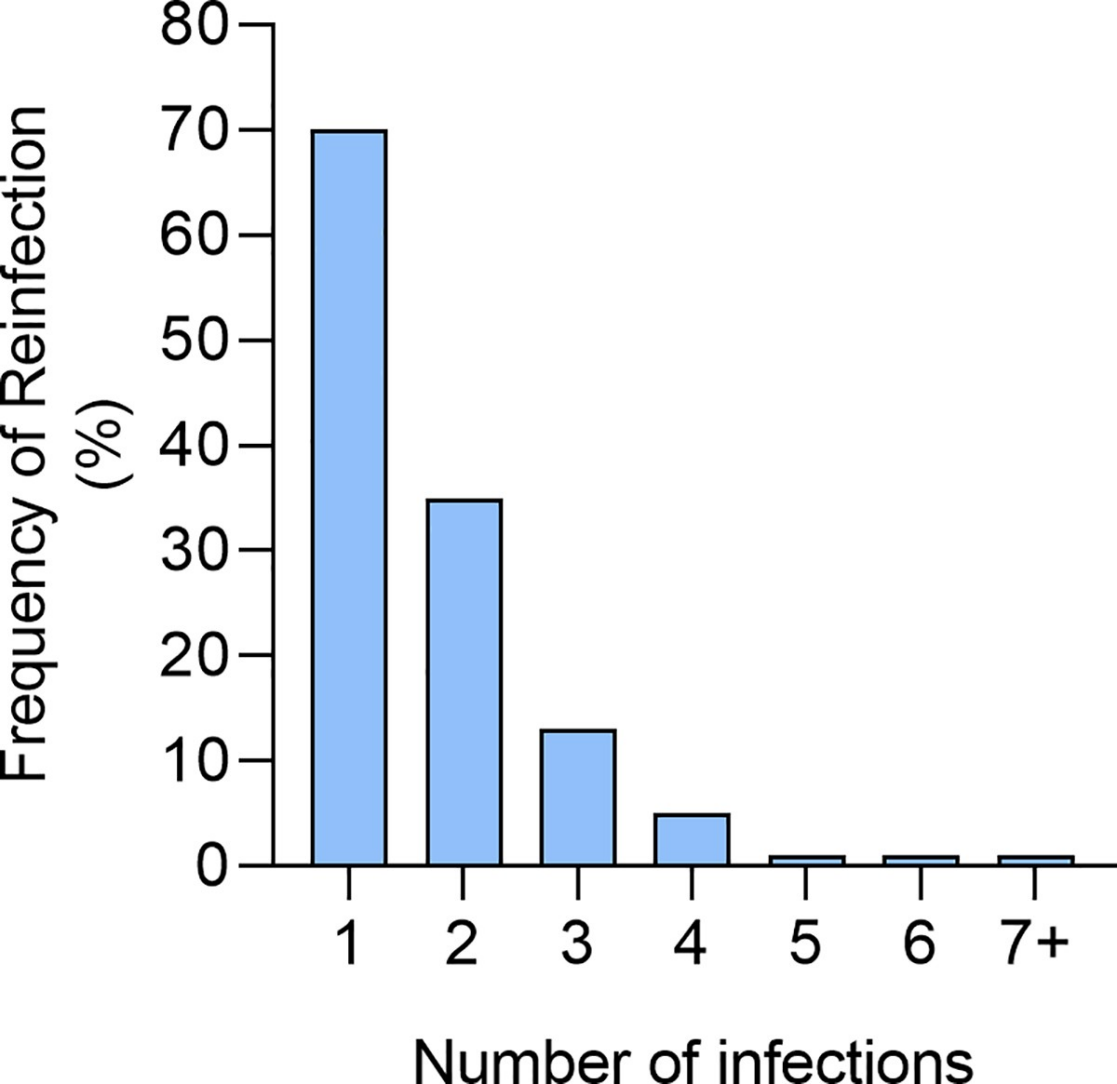
Frequency of reinfections with bilharzia among the surveyed pupils.

To determine if the national control program had impacted on reinfection, we asked the 126 children who had self-diagnosed as being positive for schistosomiasis based on haematuria and had received treatment for schistosomiasis, to indicate how many total episodes of schistosomiasis they had experienced after they had received treatment through the national control program in the past 7 years. While the pupils reported that they learned how to avoid contracting schistosomiasis as indicated in Fig 6 above, 70% (57) of the pupils had at least one reinfection with schistosomiasis after the first treatment (Fig 7).

**Fig 8:**
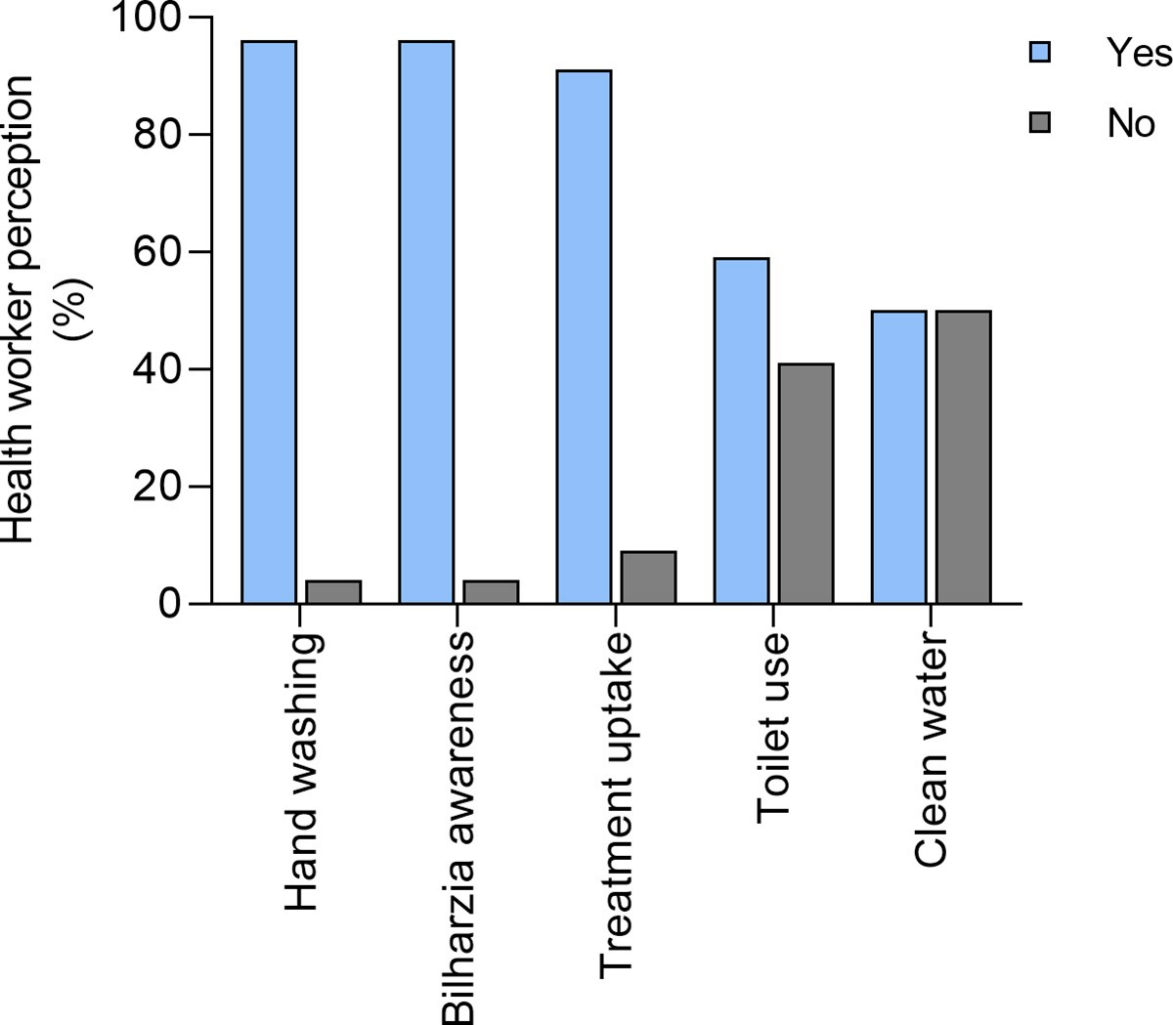
Village health worker perception on the effect of the national helminth control program on water and sanitation indicators within the community.

We determined if the impact of the national control program on KAP extended into the community by questioning the VHW. Here we aimed to determine if the KAP learnt by the children through participating in the MDAs had filtered into the children’s communities. The village health workers indicated that there had been an improvement in knowledge, behaviours and practices in the homes of the pupils. They attributed this positive change to the impact of the control program. Specifically, more than 90% of the VHW reported improvement in handwashing behaviour, schistosomiasis awareness and treatment uptake at the local clinic in the population. Of the 22 VHWs, 59% reported a noticeable improvement in toilet use in their community while only 50% of the VHW reported notable improvement in clean water use (Fig 8).

### Impact of national helminth control program on health-seeking behaviour

The village health workers are the health custodians within the community and are aware of when members of their community visit the local clinic. We, therefore, asked for their perception of their community children’s visits to the clinic for treatment for any illness. Their responses revealed that the majority of VHW 77% (17) observed a reduction in clinic visits following the national helminth control program. However, 23% (5) indicated that the pupils’ clinic visits had not changed during the 7 years of the national control program.

As students report to the school when they have to go to the local clinic during school, we asked the school teachers if the national control program improved health-seeking behaviour for the treatment of schistosomiasis among pupils. 78% (28) of the teachers perceived that the control program had empowered pupils to seek treatment once they self-diagnosed for schistosomiasis based on hematuria. Thus, whilst pupil visits to the local clinic for general ill health decreased according to the VHW, pupil health-seeking behaviour for schistosomiasis treatment in between MDAs increased.

## Discussion

Community perceptions of public health interventions, especially those that require repeat treatments are critical for adherence and therefore program success. Thus, assessment of the community perception is an important monitoring and evaluation tool. This study focused on the perceptions of the target population of a national helminth control program on the impact of the program at the individual and community level. We determined the perception of Zimbabwean primary school children, who had been treated annually between 2012 and 2017, as well as that of their schoolteachers and VHW serving their communities, who were involved in the implementation of the control program.

Unlike other control programs which use community members e.g. community health workers, in Zimbabwe’s MDA program, treatment was given by registered nurses and all treatments were recorded in the school’s MDA register. This avoided some of the problems that have been reported from other studies of treatment biases towards influential, well-embedded individuals in friendship networks [11], or misconceptions regarding treatment and mistrust of the treatments [12].

The survey indicated that 84% (342) of the children responded that their awareness of schistosomiasis had improved because of participating in the MDAs. This is significantly higher than the proportions of similar age children knowing about schistosomiasis from other studies e.g. Tanzania reported that only 17% of the children knew about schistosomiasis [13]. Of the four different aspects of schistosomiasis in the community; i.e. transmission, disease, treatment and infection avoidance, the largest proportion of children indicated that their understanding of how to avoid contracting schistosomiasis had improved, e.g. avoiding contact with freshwater sources. The study, however, did not access whether the children had reduced or changed the behaviours that exposed them to infection. Nonetheless, improved knowledge has been shown to be important for compliance to treatment. In a study along the shores of Lake Victoria area in Kenya, knowledge on the MDA program and disease were the strongest predictors of compliance/non-compliance to schistosomiasis treatment (https://repository.maseno.ac.ke/handle/123456789/1235) and in other continents [14].

Despite the knowledge on schistosome transmission, some children were reinfected with schistosomiasis over the 7 years, for example among the children who self-diagnosed with schistosomiasis, 70% reported at least one re-infection. Reinfections are not uncommon in areas of high schistosome transmission such as this one [6]. There is a greater chance of individuals being infected with immature schistosomes at the time of MDA treatment and parasites not being completely cleared by one dose of praziquantel [15]. Furthermore, high levels of reinfection can occur especially if the MDA occurs before the transmission period. Treatment of only school children excluding adults and preschool children potentially maintains transmission. All of these factors may explain the observed reinfection rate [16, 17]. Further, treatment without improving the water, sanitation and hygiene (WASH) provision does not stop transmission of the parasites [18]. Some of the children still have to cross schistosome-infective rivers to get to and from school and others still have to collect river water to water plants in the family vegetable gardens. Thus, the reinfection is consistent with the reported perception of the VHW at community level that while more than 90% of the VHW reported improvement in handwashing behaviour, schistosomiasis awareness behaviour and treatment uptake within the community, 59% of the VHW reported improvement in toilet use and only 50% of the VHW reported improvement in clean water use within their communities. This underlines the importance of integrating WASH improvements in provision of safe water and sanitation as well as adherence/compliance for long-term sustainable interventions for schistosomiasis as has been described in other studies [18–22]. Given the passive KAP gains made during the national control program, having an active and focused awareness campaign as part of the national control program might augment the KAP gains of the communities at risk of schistosome infection. The youth voice is coming to the forefront in the fight against NTDs (see https://www.youthcombatingntds.org/). Thus, the fact that knowledge gathered by the children at school could trickle down to the families to improve WASH means there is need to more effectively leverage the students as agents of change for helminth control in the country.

While the VHW reported a decline in clinic visits among the schoolchildren, for general ill health, the schoolteachers reported an increase in self-referrals after self-diagnosis of schistosomiasis among the children. The integration of schistosome control in primary health centres is effective [23] and a possible long-term replacement of MDA programs. Thus, having a community sensitized to seek treatment at the primary health centre would be an advantage. The VHW report of reduced clinic visits for general ill health are consistent with results from our cohort study on the effect of the MDAs from 2012–2017 on schistosome infection and morbidity levels in school children who were treated for schistosomiasis (submitted).

To reduce the impact of poor recall, we asked the children to indicate if they had self-diagnosed with schistosomiasis during the period between 2017 (before the 2017 MDA) and the current study. 151 children indicated that they had self-diagnosed for schistosomiasis. Of these 126 had received treatment either through the 2017 MDA (the majority) or via self-referral to the local clinic. These children indicated several benefits they perceived to have been due to treatment of schistosomiasis. This included resolution of predominantly all health symptoms they identified, including haematuria, painful urination, sore stomach, tiredness and falling asleep during class lessons, although the latter two were less frequent before treatment. Both the pupils and the school authorities perceived that school attendance had improved as a result of the helminth treatment program and the teachers further perceived that the school performance of their pupils had also improved since the implementation of the national control program. This is consistent with findings from a recent systematic review using data predominantly from Africa, which reported that compared to uninfected or children treated with praziquantel, the presence of *Schistosoma* infection was associated with deficits in school attendance as well as school performance in school aged children [24].

The majority of the 151 children self-diagnosing with schistosomiasis reported that they suffered side effects following treatment. These children were likely to have been carrying heavy infections for two reasons; first they reside in a high transmission area [6] and second, they were showing clinical signs of schistosomiasis e.g. haematuria usually associated with heavy infection. Therefore, it is not surprising that they would suffer side effects of PZQ treatment as this has been reported to be due to heavy infections [25, 26]. A large double blind placebo controlled study showed that headache and abdominal pain after praziquantel treatment were more common in children with documented schistosomiasis than in schistosome-negative subjects [26]. This study also showed that the frequency and severity of side effects was related to pretreatment infection intensity. A study from Ethiopia in 6–22 year olds reported the age group with the highest frequency of side effects as 10–14 year olds, the most heavily infected aged group [25] which overlaps with the age group in our study and similar to our study. In our study, the frequency of abdominal pain as a side effect was 45%, which is comparable to the 40% reported in the Ethiopian study. Despite the PZQ side effects reported throughout the country, compliance was high and mean treatment coverage across all the provinces increased gradually from 48% in the 2012 MDA peaking at 90.3% in the cohort during the 5th MDA in 2016 [9].

There are several limitations of the study. The study was assessing the perception of a 6 year national control program two years after the last MDA. This introduced recall bias. We reduced the impact of this in two ways; first by focusing on children who had been present for all 6 MDAs, i.e. children now in 6^th^ and 7^th^ grade, and second, by restricting specific questions to the last MDA in 2017 and the intervening period between that MDA and the present study for specific questions on infection and morbidity.

There was a lack of objective records to confirm responses. Records such as attendance school registers would have allowed analysis of the attendance data, summary school records on academic performance e.g. class averages and national grade 7 exams performance would have allowed comparison of school performance from pre 2012 to the MDA years. Nonetheless, as compliance is likely to be influenced more by personal perception than objective data, these findings are informative. Finally, data on the number of people attending the local clinic treatment for schistosomiasis treatment would also have been informative. The lack of such records was beyond our control, hence where appropriate, we asked similar questions to the pupils and the adults to cross validate the responses.

### Conclusions

Regardless of these limitations, the study yielded several public health messages, which are important for the country’s future helminth control strategy. First, the study indicates that the children treated in the MDA perceived that the national control program had improved their health, attendance, school performance and awareness of schistosomiasis. The school teachers were in agreement with the children that schistosome treatment had improved the children’s school attendance and performance. Second, the VHW had perceived that the national control program had improved the community KAP. Third, the study confirmed that reinfections are still occurring in treated children indicating that exposure and transmission are ongoing. This highlights the need for an integrated approach to schistosomiasis control, particularly improvements in WASH and for further studies into reasons for reinfections as well as the need for for partnership between the affected communities and ministries of health to ensure ownership and sustainability of interventions and impact on health. Overall, the perception from the pupils, teachers and VHW in this study area is that Zimbabwe’s national helminth control program has had a positive impact on schistosomiasis disease and KAP. This positive perception is important for continued compliance, which is required to eliminate helminths as public health problems [27].
